# *In vivo* differentiation of induced pluripotent stem cells into neural stem cells by chimera formation

**DOI:** 10.1371/journal.pone.0170735

**Published:** 2017-01-31

**Authors:** Hyun Woo Choi, Yean Ju Hong, Jong Soo Kim, Hyuk Song, Ssang Gu Cho, Hojae Bae, Changsung Kim, Sung June Byun, Jeong Tae Do

**Affiliations:** 1 Department of Animal Biotechnology, College of Animal Bioscience and Technology, Konkuk University, Gwangjin-gu, Seoul, Republic of Korea; 2 Department of Cell and Developmental Biology, Max Planck Institute for Molecular Biomedicine, Münster, Germany; 3 Department of Bioindustrial Technologies, College of Animal Bioscience and Technology, Konkuk University, Gwangjin-gu, Seoul, Republic of Korea; 4 Department of Bioscience and Biotechnology, Sejong University, Gwangjin-gu, Seoul, Korea; 5 Animal Biotechnology Division, National Institute of Animal Science, RDA, Iseo-myeon, Wanju-gun, Jeollabuk-do, Korea; Johns Hopkins School of Medicine, UNITED STATES

## Abstract

Like embryonic stem cells, induced pluripotent stem cells (iPSCs) can differentiate into all three germ layers in an *in vitro* system. Here, we developed a new technology for obtaining neural stem cells (NSCs) from iPSCs through chimera formation, in an *in vivo* environment. iPSCs contributed to the neural lineage in the chimera, which could be efficiently purified and directly cultured as NSCs *in vitro*. The iPSC-derived, *in vivo*-differentiated NSCs expressed NSC markers, and their gene-expression pattern more closely resembled that of fetal brain-derived NSCs than *in vitro*-differentiated NSCs. This system could be applied for differentiating pluripotent stem cells into specialized cell types whose differentiation protocols are not well established.

## Introduction

Pluripotent stem cells are currently considered a valuable resource for studying regenerative biology and medicine due to their tremendous differentiation potential into all cell types within the body. To be physiologically relevant, cells differentiated from pluripotent stem cells in vitro should behave very similarly to their *in vivo* counterparts in both molecular and functional terms. Therefore, developing proper protocols for differentiating pluripotent stem cells into specific cell types is a critical step for studying developmental biology and advancing applications to the clinical stage. For these purposes, long-term expandable somatic cell types have been derived from pluripotent stem cells, including embryonic stem cell (ESC)- or induced pluripotent stem cell (iPSC)-derived neural stem cells (NSCs) [1–3].

Neural stem cells (NSCs) are self-renewing multipotent stem cells that can differentiate into neurons, astrocytes, and oligodendrocytes [4]. Thus, NSCs can potentially aid the study of neural development/differentiation and various neurodegenerative disorders [5]. NSCs were initially derived and maintained *in vitro* as 3-dimensional (3D) aggregates known as neurospheres [6–8], which are relatively heterogeneous cell populations showing graduated developmental stages of neural subtypes [9–11]. Defined adherent 2D cultures, which enable the continuous expansion of pure NSC populations, were established by adding growth factors, such as fibroblast growth factor 2 (FGF2) and epidermal growth factor (EGF), to the culture media [2]. Recently, Waele et al. developed a new in vitro NSC culture system using decellularized mouse brain sections, which support the long-term culture of undifferentiated NSCs [12]. However, in vitro NSC populations in neurospheres and adherent cultures did not faithfully represent the properties of NSCs *in vivo* [7], as the *in vivo* NSC niche is the most complex system of the body and is yet to be fully understood [13]. Thus, in vitro NSCs cannot fully recapitulate *in vivo* system.

Here, we developed a new approach for differentiating NSCs that is based on the chimera-forming ability of iPSCs. Chimera formation is one of the most stringent assay to test functional pluripotency of *in vivo* embryonic cells or *in vitro* expanded pluripotent stem cells. When pluripotent stem cells are injected into a normal blastocyst, they become incorporated into the inner cell mass (ICM) and form a chimeric blastocyst, which develops into a chimeric embryo after transfer to a surrogate mother. Naïve pluripotent stem cells should form a chimera, which contains cells of 2 different origins (the blastocyst and injected pluripotent stem cells), in various tissue types, including endodermal, ectodermal, and mesodermal tissues. In this study, iPSCs successfully contributed to the brain tissue of chimeric embryos, from which iPSC-derived NSCs could be isolated and cultured. The *in vivo* NSCs derived from chimeric brain tissue were very similar to fetal brain-derived NSCs and, thus, were further characterized.

## Materials and methods

### Animal use ethical statement

Experiments were carried out in accordance with the approved guidelines and all experimental protocols were approved by the Institutional Animal Care and Use Committee (IACUC) of Konkuk University. All mouse strains were bred and housed at the mouse facility of the Konkuk University or were bought from Orient-Bio Inc. (Gyeonggi-do, Korea; http://www.orient.co.kr). Animal welfare was under control of local committees. Mice were housed in a temperature-controlled room with automated darkness-light cycle system, fed with a regular ad libitum feeding. Before oocyte harvesting, mice were sacrificed by carbon dioxide inhalation.

### Generation and culture of iPSCs

iPSCs were generated using the same protocol reported previously [14]. MEFs obtained from OG2^+/-^/ROSA26^+/-^ double-transgenic mice which were carrying *Oct4*-*GFP* and *neo*/*lacZ* were infected with retroviruses encoding 4 transcription factors (*Oct4*, *Sox2*, *Klf4*, and c-*Myc*). After retroviral infection, Oct4-GFP-positive colonies were transferred onto inactivated MEF feeder layers, trypsinized, re-plated, and cultured in ESC medium (Dulbecco's modified Eagle's medium (DMEM; Gibco) supplemented with 15% fetal bovine serum (FBS; Gibco), 1× penicillin/streptomycin/glutamine, 1 mM nonessential amino acids (NEAA; Gibco), 0.1 mM β-mercaptoethanol (Gibco), and 1000 U/ml leukemia inhibitory factor (LIF; ESGRO, Chemicon) [14].

### Generation and culture of fetal brain-NSCs

To derive Fetal brain-NSCs, brain tissue was collected from 13.5 days postcoitum (dpc) fetuses, which were ROSA26/OG2 heterozygous double transgenic. Neurospheres cultured from brain tissue were prepared as described in detail in our previous article [15]. In Brief, brain tissue was enzymatically dissociated in HBSS (with 2 mM glucose) containing 0.7 mg/ml hyaluronic acid, 0.2 mg/ml kynurenic acid, and 1.33 mg/ml trypsin at 37`C for 30 min. The dissociated cells were passed through a 70um nylon mesh to remove large cell clusters. The cells were then centrifuged at 200 g for 5 min and collected by centrifugation in 0.9 M sucrose in 0.5× HBSS at 750 g for 10 min. The cell pellet was resuspended in 2 ml of culture medium, placed on top of 10 ml of 4% bovine serum albumin (BSA) in EBSS solution, and centrifuged at 200 g for 7 min. The culture medium was supplemented with 20 ng/ml epidermal growth factor (EGF; Gibco BRL), 20 ng/ml basic fibroblast growth factor (bFGF), B27 supplement (Gibco BRL), 8 mM HEPES, 2 mM glutamine, 100 U/ml penicillin, and 100 mg/ml streptomycin in DMEM-F12 medium (Gibco BRL). Primary neurospheres were replated onto gelatinized dishes in NSC expansion medium: NS-A media (Euroclone) supplemented with N2 supplement, 10 ng/ml of EGF, and bFGF (Invitrogen), 50 mg/ml bovine serum albumin (BSA Fraction V; Gibco BRL), 1× penicillin/streptomycin/glutamine, and 1 × nonessential amino acids (Gibco BRL). Outgrowing cells were trypsinized, replated, and cultured in NSC expansion medium. NSCs were established by dissociation and replated onto gelatin-coated dishes in NSC expansion medium.

### Differentiation of iPSCs into NSCs *in vitro* and *in vivo*

For *in vitro* differentiation, iPSCs (passage 22) were cultured for 2–3 days with MEF medium supplemented with 15% FBS, 1× penicillin/streptomycin/glutamine, 0.1 mM non-essential amino acids, and 1 mM β-mercaptoethanol in Dulbecco’s Modified Eagle’s Medium (DMEM). After 2–3 days in culture, the ESCs were cultured for an additional 2 days without feeder cells in suspension culture dishes containing N2B27 medium (1:1 mixture of DMEM/F-12 medium [Gibco BRL] and Neurobasal Medium [Invitrogen], which was supplemented with EGF [10 ng/ml; Gibco BRL], basic fibroblast growth factor [bFGF; 10 ng/ml; Invitrogen], N2 supplement, B27 supplement [Gibco BRL], and 1× penicillin/streptomycin/glutamine). Then, the cells were plated in 0.1% Gelatin-coated dishes containing NSC expansion medium: DMEM/F12 supplemented with N2 supplement, EGF (10 ng/ml), bFGF (10 ng/ml), bovine serum albumin (BSA; 50 mg/ml; Gibco BRL), and 1× penicillin/streptomycin/glutamine; this step was followed by culture for 3 days. For *in vivo* differentiation, Clumps of iPSCs (4–10 cells, passage 22) were aggregated with denuded, post-compacted, 8-cell-stage B6C3F1 embryos to obtain aggregated embryo. The aggregated blastocysts were transferred into a uterine horn of 2.5-dpc pseudopregnant recipients. After 10 days, the whole brain from each chimeric embryo was trypsinized and dissociated into single-cell suspensions. To form neurospheres, these cells were cultured in N2B27 medium for 8–10 days. The formed neurospheres were transferred to Gelatin-coated dishes in NSC expansion medium.

### Single cell clonal expansion of NSCs

To generate single cell-derived NSC line, single cells of each NSC line were plated onto the 96well-round-bottom plate with culture medium. The culture medium was supplemented with 20 ng/ml epidermal growth factor (EGF; Gibco BRL), 20 ng/ml basic fibroblast growth factor (bFGF), B27 supplement (Gibco BRL), 8 mM HEPES, 2 mM glutamine, 100 U/ml penicillin, and 100 mg/ml streptomycin in DMEM-F12 medium (Gibco BRL). Single cells were expanded for 14 days on average. Expanded neurospheres were replated onto gelatinized dishes in NSC expansion medium: NS-A media (Euroclone) supplemented with N2 supplement, 10 ng/ml of EGF, and bFGF (Invitrogen), 50 mg/ml bovine serum albumin (BSA Fraction V; Gibco BRL), 1× penicillin/streptomycin/glutamine, and 1 × nonessential amino acids (Gibco BRL). Outgrowing cells were trypsinized, replated, and cultured in NSC expansion medium. Single cell derived-NSC lines were established by dissociation and replated onto gelatin-coated dishes in NSC expansion medium.

### Differentiation of NSCs into neurons and glial cells

For neuronal differentiation, NSCs were differentiated in N2B27 medium (1:1 mixture of DMEM/F12 medium; (Gibco), Neurobasal medium (Gibco) supplemented with N2 supplement, B27 supplement (Gibco), 1× penicillin/streptomycin/glutamine, and bFGF (10 ng/ml)) for 3days. NSCs were further differentiated for additional 8 days in N2B27 medium (1:1 mixture of DMEM/F12 medium (Gibco), Neurobasal medium (Gibco) supplemented with N2 supplement, B27 supplement (Gibco BRL), 1× penicillin/streptomycin/glutamine, and ascorbic acid (200uM)). For glial differentiation, NSCs were differentiated for 2 weeks in glial differentiation medium (1:1 mixture of DMEM/F12 medium (Gibco), Neurobasal medium (Gibco) supplemented with 1% fetal bovine serum (Gibco), N2 supplement (Gibco), B27 supplement (Gibco), 1 × penicillin/streptomycin/glutamine (Gibco)).

### X-gal staining

Chimeric embryos and chimera-derived NSCs cells were rinsed with phosphate-buffered saline (PBS) and fixed in paraformaldehyde (4%) for 20 min at 4°C. Cells were rinsed 3 times at room temperature in PBS containing ethylene glycol tetraacetic acid (5 mM), doxycholate (0.01%), NP40 (0.02%), and MgCl_2_ (2 mM). Cells were washed with PBS and stained in X-gal staining solution: PBS supplemented with 5-bromo-r-chloro-3-indolyl-galactosidase (X-gal; 1 mg/mL; Promega), K_2_Fe(CN)_6_ (5 mM), K_4_Fe(CN)_6_ (5 mM), and MgCl_2_ (1 mM). Blue staining was visualized by light microscopy.

### Immunocytochemistry

For immunocytochemistry, cells were fixed in paraformaldehyde (4%) for 20 min at 4°C. After washing with PBS, the cells were treated with PBS containing BSA (3%) and Triton X-100 (0.03%) for 45 min at room temperature. The primary antibodies used were anti-Nestin (Nestin; monoclonal, 1:500, Millipore, MAB353) anti-Sox2 (Sox2; polyclonal, 1:500, Millipore, AB5603), anti-MAP2 (MAP2; polyclonal, 1:200, Cell signalling, 4542S), and anti-GFAP (GFAP; polyclonal, 1:1000, abcam, ab7260). For detection purposes, fluorescently labelled (Alexa Fluor 488 or 568; Molecular Probes, Eugene, OR, USA) secondary antibodies were used, according to the specifications of the manufacturer.

### PCR

The PCR primers used were as follows: Oct4 TG sense 5'-GACGGCATCGCAGCTTGGATA-3', Oct4 TG antisense 5'-CCAATACCTCTGAGCCTGGT-3'; Sox2 TG sense 5'-GACGGCATCGCAGCTTGGATA-3', Sox2 TG antisense 5'-CGCTTGGCCTCGTCGATGAA-3'; Klf4 TG sense 5'-GACGGCATCGCAGCTTGGATA-3', Klf4 TG antisense 5'-GGGAAGTCGCTTCATGTGAG-3'; c-Myc TG sense 5'-GACGGCATCGCAGCTTGGATA-3', c-Myc TG antisense 5'-ACCGCAACATAGGATGGAGA-3'; and *Oct4*-GFP sense 5'-GCAAGCTGACCCTGAAGTTCA-3', *Oct4*-GFP antisense 5'-TCACCTTGATGCCGTTCTTCT-3'.

### RNA isolation and real-time (q) RT-PCR analysis

Total RNA was isolated using the RNeasy Mini Kit (Qiagen, Venlo, Netherlands, http://www.qiagen.com) and was treated with DNase to remove genomic DNA contamination. One microgram of total RNA was reverse-transcribed with Super-Script III Reverse Transcriptase Kit (Invitrogen) and oligo(dT) primer (Invitrogen) according to the manufacturer’s instructions. Quantitative polymerase chain reaction (PCR) reactions were set up in duplicate with the Power SYBR Green Master Mix (Takara) and analyzed with the Roche LightCycler 5480 (Roche). The primers for qRT-PCR used were as follows: Nestin (endo) sense 5'-AAG TTC CCA GGC TTC TCT TG-3', Nestin (endo) antisense 5'-GTC TCA AGG GTA TTA GGC AAG G-3'; Sox2 (endo) sense 5'-CAT GAG AGC AAG TAC TGG CAA G-3', Sox2 (endo) anti-sense 5'-CCA ACG ATA TCA ACC TGC ATG G-3', Oct4 TG sense 5’-GACGGCATCG CAGCTTGGATA-30, Oct4 TG antisense 5’-CCAATACCTCTGAGCCTGGT-3’; Sox2 TG sense 50-GACGGCATCGCAGCTTGGATA-30, Sox2 TG antisense 5’-CGCTTGGCCTCGTCGATGAA-30; Klf4 TG sense 5’-GACGGCATCGCAGCTTGGATA-3’, Klf4 TG antisense 5’-GGGAAGTCGCTTCATGTGAG-3’; c-Myc TG sense 5’-GACGGCATCGCAGCTTGGATA-30, c-Myc TG antisense 50-ACCGCAACATAG GATGGAGA-30; ACTB sense 5’-CGCCATGGATGACGATATCG-3’, and ACTB antisense 5’-CGAAGCCGGCTTTGCACATG-3’.

### Microarray

Fetal brain-NSCs (from 13.5dpc fetuses, ROSA26/OG2 heterozygous double transgenic), iPS-NSCs #1, #3 (In vitro differentiated), iPS-cNSCs (In vivo differentiated, chimeric), NED-NSCs (normal embryo-derived), MEFs (from 13.5dpc fetuses, ROSA26/OG2 heterozygous double transgenic), and ESCs (OG2 heterozygous double transgenic) were used for analysis. Total RNA was isolated using the RNeasy Mini Kit (Qiagen) and digested with DNase I (RNase-free DNase, Qiagen), according to the manufacturer’s instructions. Total RNA was amplified, biotinylated, and purified using the Ambion Illumina RNA Amplification Kit (Ambion), according to the manufacturer’s instructions. Labelled cRNA samples (750 ng) were hybridized to MouseRef-8 v2 Expression BeadChip. Signal detection was performed with Amersham Fluorolink Streptavidin-Cy3 (GE Healthcare Bio-Science), according to the bead array manual. Arrays were scanned with an Illumina Bead Array Reader, according to the manufacturer’s instructions.

Raw data were extracted using the software provided by the manufacturer (Illumina GenomeStudio v2011.1, Gene Expression Module v1.9.0). Array data were filtered using a detection *p* value < 0.05 in at least 50% of the samples. Selected probe signals were log-transformed and normalized using the quantile method. Comparative analysis was performed using the local-pooled-error test and fold-change. The false discovery rate was controlled by adjusting the *p* value with the Benjamini–Hochberg algorithm. Hierarchical clustering was performed using complete linkage and the Pearson distance as a measure of similarity. MDS analysis was performed using expression data of sample. Distance matrix within sample was calculated by Euclidean method. Caculation was performed with classical multidimensional scaling. The gene expression profiling files are available from the GEO database (accession number GSE87597)

## Results

### Differentiation of iPSCs into NSCs *in vivo* by chimera formation

**Fig 1:**
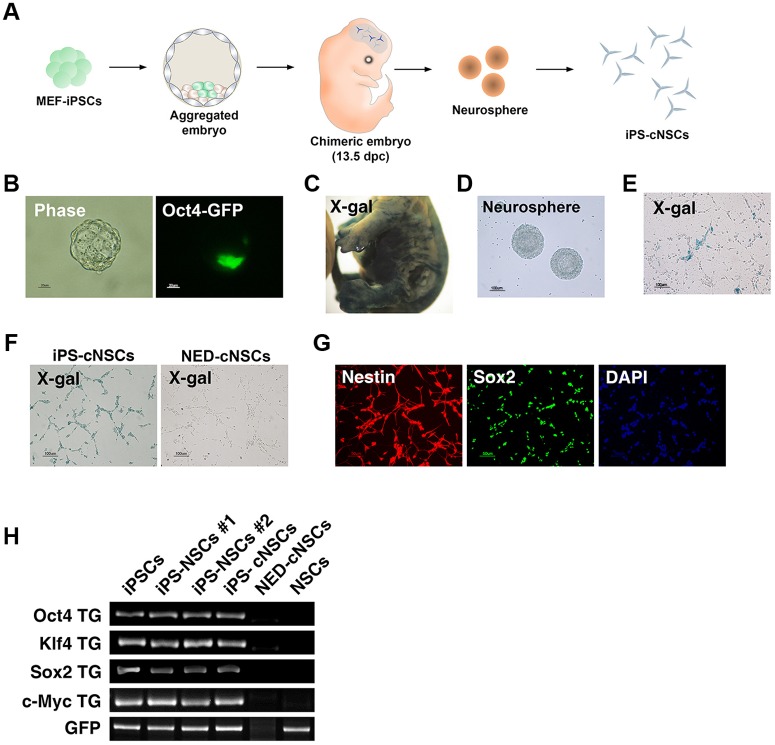
*In vivo* differentiation of iPSCs into NSCs after chimera formation. **(A)** Scheme of *in vivo* differentiation of iPSCs into NSCs. **(B)** Aggregated embryo with iPSCs. iPSCs could incorporate into the ICM (Oct4-GFP^+^). **(C)** X-gal staining of chimeric embryos (iPSCs). **(D)** Neurosphere formation and **(E)** 2D culturing of NSCs derived from a chimeric embryo. X-gal-positive staining of *in vivo*-differentiated NSCs derived from iPSCs. **(F)** X-gal staining of iPS-cNSCs and NED-cNSCs. iPS-cNSCs were positive for X-gal staining, but NED-cNSCs were not. **(G)** iPS-cNSCs expressed NSC markers, such as NESTIN and SOX2, as determined by immunocytochemistry. **(H)** Genotyping of transgenes in iPSCs, iPS-NSCs (#1 and #2), iPS-cNSCs, NED-cNSCs, and control NSCs (OG2^+/-^/ROSA26^+/-^). iPSCs and iPS-derived cells contained transgenes.

First, we generated iPSCs using murine embryonic fibroblasts (MEFs) obtained from OG2^+/-^/ROSA26^+/-^ double-transgenic mice. MEFs were infected with retroviruses encoding 4 transcription factors, namely *Oct4*, *Sox2*, *Klf4*, and c-*Myc*. The pluripotency of these iPSC lines was confirmed previously, as was the expression of pluripotency markers, reactivation of the inactive X chromosome, and formation of germline chimera [14]. We endeavoured to differentiate iPSCs into NSCs using an *in vivo* system (Fig 1A). We utilized the fact that ESCs or iPSCs could form a chimera after blastocyst injection or morula aggregation followed by transfer into a surrogate mother [16, 17]. ESCs or iPSCs can contribute throughout the whole body of a chimera, including the germ cells. To distinguish the cellular origin (embryonic or pluripotent cells), it is essential to use iPSCs that express selection markers. As iPSCs were derived from MEFs containing *Oct4-GFP* and *neo*/*lacZ* in the *Rosa* locus, they expressed GFP in the pluripotent state and ubiquitously expressed the *neo*/*lacZ* transgene. Thus, differentiated cells from these iPSCs lose GFP expression, but maintain *neo*/*lac Z* expression, which confers them with neomycin resistance and positive X-gal staining [15, 18].

**Fig 2:**
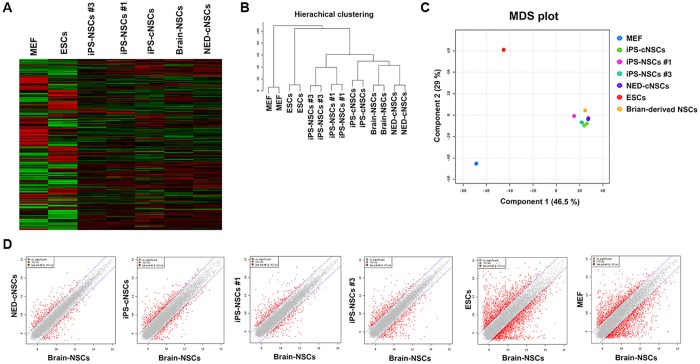
Gene-expression pattern of iPS-derived NSCs, in vitro and in vivo. **(A)** Heatmap analysis of MEFs, ESCs, iPS-NSCs (#1 and #3), iPSC-cNSCs, brain-derived NSCs, and NED-cNSCs. **(B)** Hierarchical clustering analysis of MEFs, ESCs, iPS-NSCs (#1 and #3), iPSC-cNSCs, brain-derived NSCs, and NED-cNSCs. **(C–D)** MDS and scatter-plot analysis of MEFs, ESCs, iPS-NSCs (#1 and #2), iPSC-cNSCs, brain-derived NSCs, and NED-cNSCs.

For the *in vivo* differentiation of iPSCs, we aggregated iPSCs with morulas to form chimeric blastocysts (Fig 1B). After transferring them to a pseudo-pregnant mother, chimeric embryos were formed (Fig 1C). X-gal staining showed that iPSCs contributed to the chimeric embryo (Fig 1C). Whole brain tissue was dissected from the chimeric embryos, dissociated into single cells by trypsinization, and cultured in neurosphere medium to form neurospheres (Fig 1D). The neurospheres were re-plated into NSC medium containing neomycin. Initially, the chimeric embryo-derived NSCs contained a small population of X-gal-positive cells among mostly negative cells (Fig 1E). After culturing for 10 days in neomycin-containing NSC medium, pure populations of iPSC-derived NSCs were obtained (Fig 1F). Therefore, we could successfully established iPSC-derived NSC cell lines from chimeric embryos, which are referred to here as iPS-cNSCs (Fig 1F). We also established X-gal- negative NSCs from chimeric embryos as a negative control, which were normal embryo-derived NSCs (NED-cNSCs; Fig 2F). The iPS-cNSCs expressed neural stem cell markers such as Nestin and Sox2 (Fig 2G, S1 Fig). To determine whether iPS-cNSCs have normal differentiation potential in clonal level, we established and characterized iPS-cNSC-Single cell line (iPS-cNSC-S) from the single clone (S2 Fig). They could differentiate into neural or glial subtypes. GFAP and MAP2 staining revealed their differentiation potential is similar for NED-NSCs (S3 Fig). PCR analysis confirmed that iPSCs (passage 22), iPS-NSCs (in vitro differentiated, #1 and #2) [14], and iPS-cNSCs contained transgenes of viral origin (4 reprogramming factors) and *Oct4*-*GFP* (Fig 1H), but that NED-cNSCs did not, indicating that iPSC-derived cells and NED cells could be successfully purified. Moreover, we confirmed these transgenes were successfully silenced in iPS-cNSCs (S4 Fig). This approach represents a new technology for differentiating iPSCs into NSCs in an *in vivo* environment. We speculate that NSCs established from iPSCs through *in vivo* differentiation systems may display different molecular characteristics and, thus, these cells were further characterized to compare the molecular signature of the *in vitro* and *in vivo* differentiation systems.

### Gene expression patterns of iPS-NSCs and iPS-cNSCs

To compare the similarity of iPS-NSCs (in vitro system) and iPS-cNSCs to the fetal brain-derived NSCs (brain-NSCs), we performed mRNA microarray analysis with MEFs, ESCs, iPS-NSCs, iPS-cNSCs, NED-cNSCs, and fetal brain-NSCs (Fig 2A–2D). The heap map and scatter plot analyses showed that all NSCs derived from iPSCs through the *in vitro* and *in vivo* systems showed similar gene-expression profiles compared to fetal brain-NSCs (Fig 2A and 2D). However, hierarchical clustering and MDS plot analyses showed small differences in the plot distributions detected between the different iPSC-derived NSCs (in vitro- and in vivo-derived). Gene-expression patterns of iPS-cNSCs (*in vivo*-derived) were closer to fetal brain-NSCs than those of iPS-NSCs (*in vitro*-derived cell lines, iPS-NSCs #1 and #3), as shown in Fig 2B and 2C. Interestingly, the gene-expression pattern of *in vivo* control NED-cNSCs was slightly more similar to that of the fetal brain-NSCs than the iPS-cNSCs (Fig 2B and 2C). These results indicated that both the differentiation environment and the cell source may influence the characteristics of their differentiation products.

### Differential gene expression in iPS-NSCs and iPS-cNSCs

**Fig 3:**
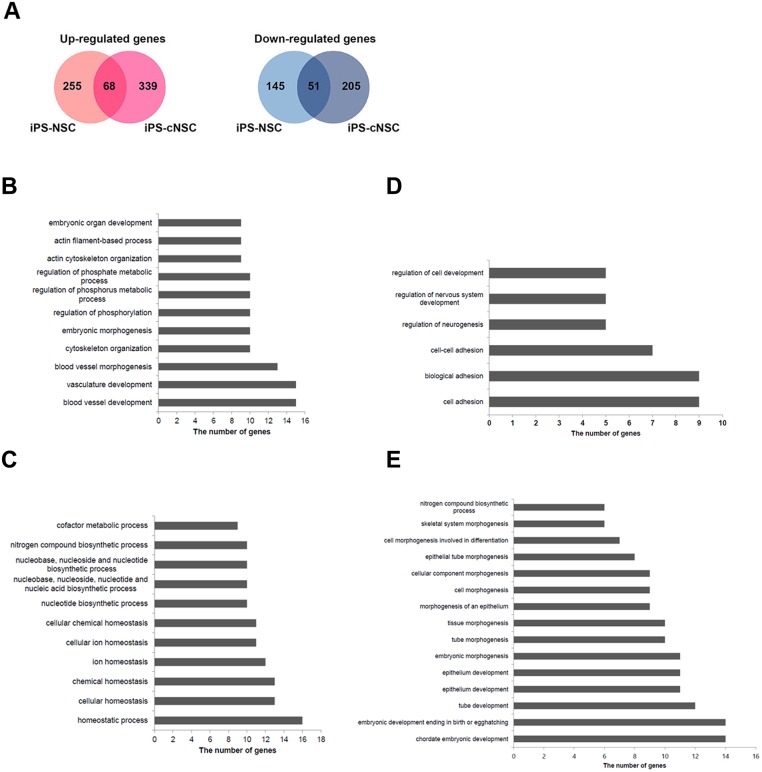
Differentially expressed genes in iPS-NSCs and iPS-cNSCs. **(A)** Up-regulated or down-regulated genes in iPS-NSCs or iPS-cNSCs, compared with the corresponding expression levels in brain-derived NSCs. **(B)** GO analysis of genes that were up-regulated in iPS-NSCs, when compared with brain-derived NSCs. **(C)** GO analysis of genes that were up-regulated in iPS-cNSCs, when compared with brain-derived NSCs. **(D)** GO analysis of down-regulated genes in iPS-NSCs. **(E)** GO analysis of down-regulated genes in iPS-cNSCs.

To analyse differentially expressed genes in iPSC-derived NSCs, we compared up-regulated genes or down-regulated genes between iPS-NSCs and iPS-cNSCs (fold-change [FC] > 2). In iPS-NSCs, 323 genes were up-regulated compared to fetal brain-derived NSCs (Fig 3A). In total, 407 genes were up-regulated in iPS-cNSCs compared to the corresponding expression levels in fetal brain-derived NSCs. In addition, 68 genes were identified as up-regulated genes in both iPS-NSCs (21%) and iPS-cNSCs (16.7%). We also identified 196 and 256 genes that were down-regulated in iPS-NSCs and iPS-cNSCs, respectively, compared to their expression levels in fetal brain-derived NSCs. Further analysis revealed that 51 genes were down-regulated in both iPS-NSCs (26%) and iPS-cNSCs (19.8%).

Next, we performed Gene Ontology (GO) analysis of the differentially expressed genes identified in iPS-NSCs and iPS-cNSCs. (S1 and S2 Tables) Functional annotation clustering of differentially expressed genes using GO analysis revealed that the up-regulated genes in iPS-NSCs were significantly enriched for GO terms linked to ‘blood vessel development’, ‘actin cytoskeleton organization’, ‘embryonic organ development’, ‘regulation phosphorylation’, ‘cholesterol and sterol biosynthetic process’, ‘membrane invagination’, and ‘regulation protein kinase activity’. The up-regulated genes in iPS-cNSCs were enriched for GO terms linked to ‘acetyl-CoA and coenzyme metabolic process’, ‘energy derivation by oxidation’, ‘nucleotide biosynthetic process’, ‘regulation of neurological system process’, ‘cellular homeostasis’, and ‘regulation of nervous system development’. Pathway analysis (KEGG pathway) revealed that up-regulated genes in iPS-NSCs were enriched for terms associated with ‘small cell lung cancer’ and ‘cardiomyopathy’. The up-regulated genes in iPS-cNSCs were enriched for terms associated with ‘oxidative phosphorylation’ and ‘Alzheimer‘s disease’ (Fig 3B and 3C, S1 Table). The down-regulated genes in iPS-NSCs were enriched for terms linked to ‘cell-cell adhesion’ and ‘regulation neurogenesis’. The down-regulated genes in iPS-cNSCs were enriched for terms linked to ‘tube development’, ‘epithelial tube morphogenesis’, ‘chordate embryonic development’, ‘neural tube formation’, ‘embryonic skeletal system morphogenesis’, ‘cell morphogenesis’, and ‘pyrimidine nucleotide biosynthetic process’ (Fig 3D and 3E, S2 Table).

## Discussion

Here, we established *in vivo* differentiated NSCs (iPS-cNSCs) from iPSCs by chimera formation, which were similar to fetal brain-derived NSCs in terms of morphology and gene-expression patterns. These results indicated that iPSCs contributed to the neural lineage in chimeras (*in vivo* environment), which could be purified and then directly cultured as NSCs *in vitro*. NSCs established from iPSCs through *in vitro* and *in vivo* differentiation systems are very similar to fetal brain-derived NSCs, but NSCs differentiated from an *in vivo* system were slightly more similar to fetal brain-derived NSCs than *in vitro*-differentiated NSCs. Araki et al. (2013) used *in vivo*-differentiated somatic cells from iPSCs for transplantations. They transplanted skin cells, a type of in vivo-differentiated somatic cells, from the tails of chimeric mice and showed that the transplanted cells were sustained over 10 months, which exceeded the period in which *in vitro*-differentiated somatic cells were sustained post-transplantation [19]. There is another useful approach for *in vivo* differentiation system. Specific cell types could be isolated from pluripotent stem cells through teratoma formation, which was based on the ideas of teratoma contained diverse cell types [20–22]. We also found that in vivo differentiation through teratoma formation could be used to differentiate PSCs into NSCs [23]. Chimera-derived differentiated NSCs also were more similar to fetal brain-derived NSCs than to NSCs derived by *in vitro* differentiation. These results suggested that the differentiation environment influences gene expression in differentiated cells and could be a crucial factor determining the characteristics of the differentiated cells from iPSCs.

Differentiating pluripotent stem cells into specialized cell types is potentially useful, not only for clinical transplantation applications, but also as a research tool for studying the basic mechanisms of diseases. Recently, pluripotent stem cells such as ESCs and iPSCs were reported to be differentiated into NSCs *in vitro*, which could be maintained in culture, while retaining the ability to differentiate into neuronal or glial cells [2, 14]. However, data from a recent study showed that the low efficiency of differentiation from iPSCs was associated with transgene reactivation [24]. Transgenes of reprogramming factors can be integrated into the genomes of iPSCs using retroviral, lentiviral, or plasmid vector systems [25]. The *in vitro* differentiation of retrovirus-derived iPSCs into NSCs was slower (4–7 weeks) than that of ESCs (1–2 weeks) [14]. Here we showed that *in vivo* differentiation following chimera formation could overcome the differentiation delay problem of retrovirus-derived iPSCs. The iPSCs developed using retroviral transgene expression efficiently differentiated into NSCs through chimera formation without a delay of NSC formation in brain tissue. Furthermore, we could not see re-reprogramming in the iPS-cNSCs, which was observed in NSCs differentiated in vitro [14].

The results of several studies have suggested that lentiviral or retroviral transgenes were re-expressed when the iPSCs were differentiated *in vitro* [14, 24]. iPSC-derived neurospheres contained undifferentiated Nanog-GFP-positive cells, which could form teratomas in NOD/SCID mice [26]. Exogenous *Oct4* expression was detected in Nestin-positive neural progenitor cells and in TuJ1- and TH-positive neurons differentiated from human iPSCs induced by a lentivirus [24]. Moreover, we showed that NSCs differentiated from iPSCs could be reverted into the pluripotent state after 10 passages. The integrated transgenes in iPSCs were reactivated after differentiation into NSCs, following the down-regulation of DNA methyltransferases and DNA methylation of the transgenes [14]. However, only about 33% (1/3) of the NSC lines reverted into pluripotent cells, indicating that not all transgenes were reactivated when iPSCs differentiated. Here, we showed that *in vivo*-differentiated NSCs did not contain undifferentiated cells (S5 Fig). We derived 2 iPS-cNSC lines, which did not show any sign of reprogramming into the pluripotent state after more than 10 passages. Moreover, exogenous factors were silenced in all iPS-cNSC cell lines (S4 Fig).

In this study, we successfully differentiated and isolated NSCs from iPSCs after chimera formation. This system could be applied to obtain various differentiated cell types from iPSCs, such as hepatocytes or endothelial cells. In addition, the *in vivo* differentiation method based on chimera formation can be used for differentiating pluripotent stem cells into cell types whose differentiation protocols have not been well established. Although ethical considerations challenge translating the chimera-formation method to humans, as a therapeutic resource, as naïve human pluripotent stem cells were established recently [27–29], there is a possibility for cross-species chimera formation. Also, an efficient differentiation protocol or mimic of the *in vivo* environment should be developed for human applications, such as with an *in vitro* 3D cerebral organoid culture system.

## Supporting information

S1 Fig Neural stem cell marker expression of iPS-NSCs and iPS-cNSCs. **(A)** Gene expression levels of the NSC marker Nestin and Sox2 in brain-derived NSCs, iPS-NSCs, and iPS-cNSCs. Data are presented as mean±SD of triplicates (n = 3).
(TIF)

S2 Fig Establishment of iPS-cNSC-single cell line (iPS-cNSC-S). **(A)** Neurosphere formation and establishment of iPS-cNSC-Single cell-line (iPS-cNSC-S) from single cell. **(B)** iPS-cNSCs expressed NSC markers, such as NESTIN and SOX2, as determined by immunocytochemistry.
(TIF)

S3 Fig Differentiation potential of NED-NSCs and iPS-cNSCs. **(A)** iPS-cNSC-S and NED-NSCs could differentiate into glial (GFAP+) or neural (MAP2+) lineage. **(B)** Quantification of the lineage-specific differentiation efficiency by NED-NSCs and iPS-cNSC-S. Error bars indicate the standard error of the mean.
(TIF)

S4 Fig Transgene expression of iPS-cNSCs. **(A)** Expression levels of exogenous 4 Factor (Oct4-TG, Klf4-TG, Sox2-TG, and c-Myc-TG) in iPSCs (Negative control), iPS-NSCs (Positive control, Transgene re-expressed, established in our previous article [14]), iPS-cNSCs, and NSCs 4F (Positive control). NSCs 4F were control, infected retroviral four factors in, respectively. Data are presented as mean±SD of triplicates (n = 3).
(TIF)

S5 Fig Inactivation of Oct4-GFP in iPS-derived NSCs. **(A)** iPS-cNSC-S and iPS-NSCs were negative for Oct4-GFP transgene expression.
(TIF)

S1 Table GO analysis and KEGG-pathway analysis of genes that were up-regulated in iPS-NSCs, when compared with brain-derived NSCs. (PDF)

S2 Table GO analysis and KEGG-pathway analysis of genes that were down-regulated in iPS-NSCs, when compared with brain-derived NSCs. (PDF)
