# Supplementary figures and images for: In vivo differentiation of induced pluripotent stem cells into neural stem cells by chimera formation
Source: PLoS One. 2017 Jan 31;12(1):e0170735. doi: 10.1371/journal.pone.0170735 (PMC5283667; doi:10.1371/journal.pone.0170735)

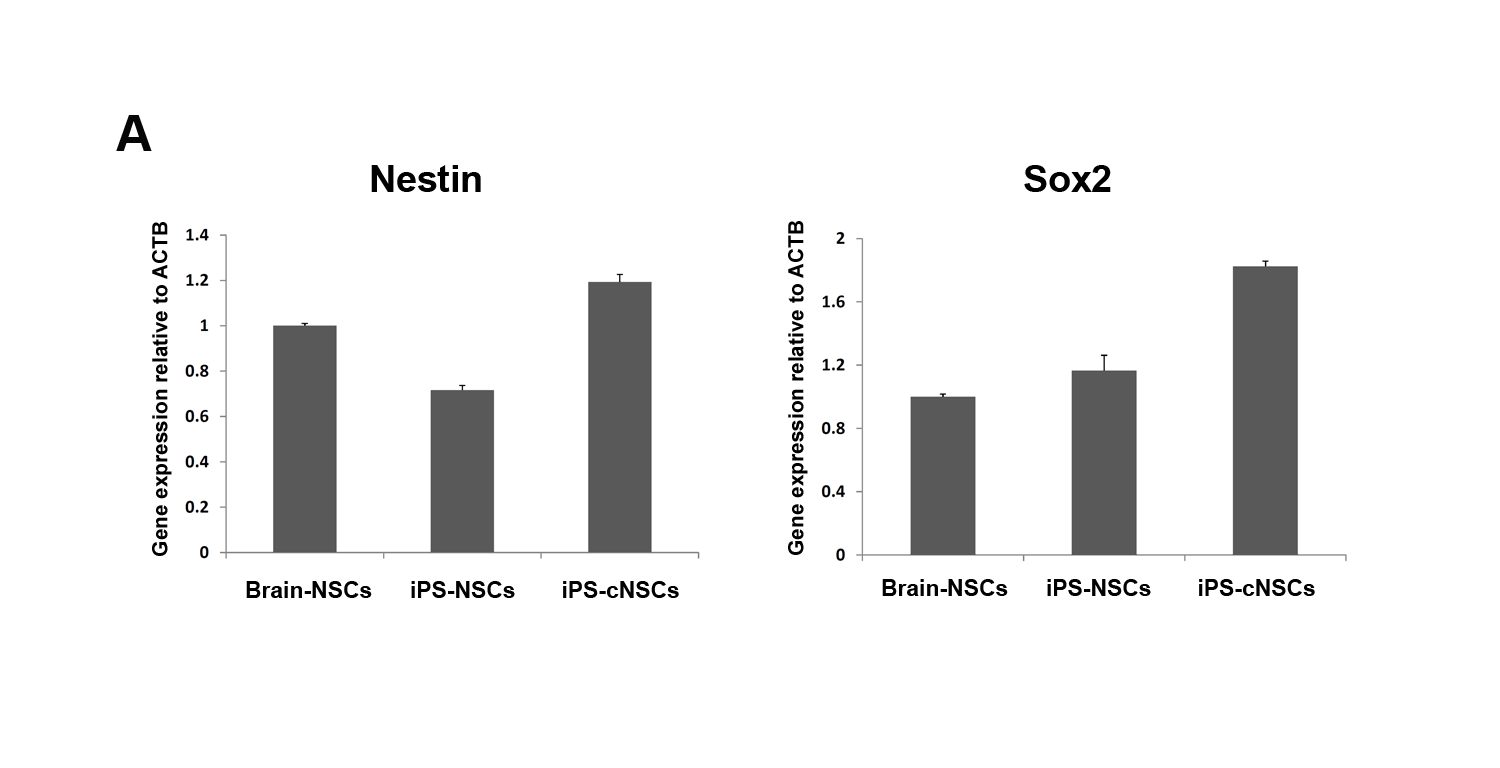

Supplement: S1 Fig — (A) Gene expression levels of the NSC marker Nestin and Sox2 in brain-derived NSCs, iPS-NSCs, and iPS-cNSCs. Data are presented as mean±SD of triplicates (n = 3). (TIF) [file pone.0170735.s001.tif]

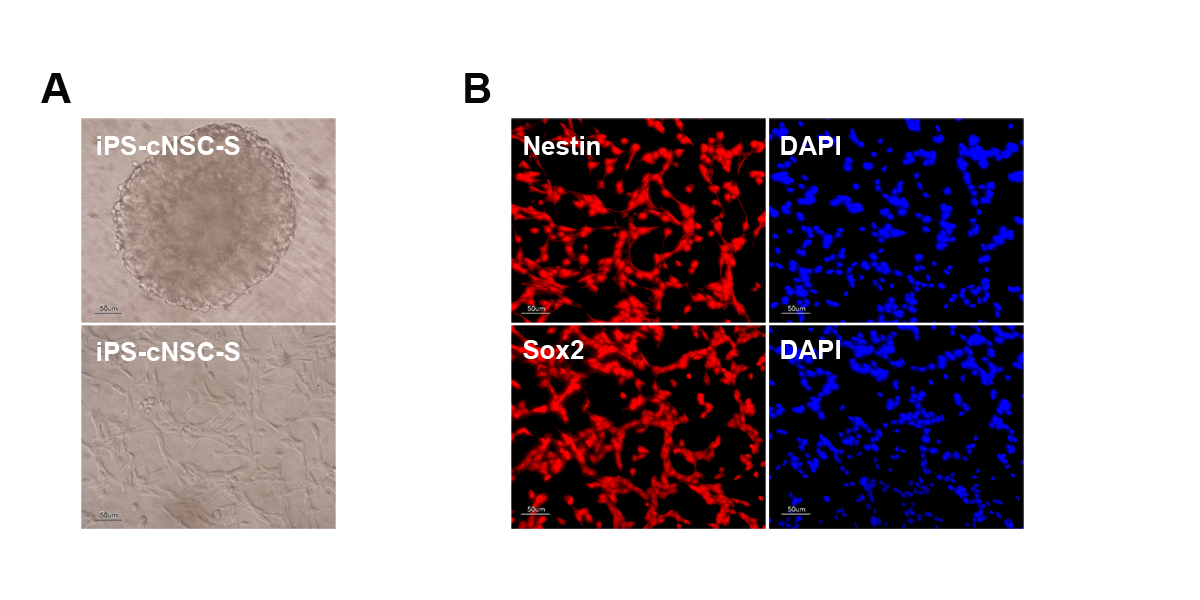

Supplement: S2 Fig — (A) Neurosphere formation and establishment of iPS-cNSC-Single cell-line (iPS-cNSC-S) from single cell. (B) iPS-cNSCs expressed NSC markers, such as NESTIN and SOX2, as determined by immunocytochemistry. (TIF) [file pone.0170735.s002.tif]

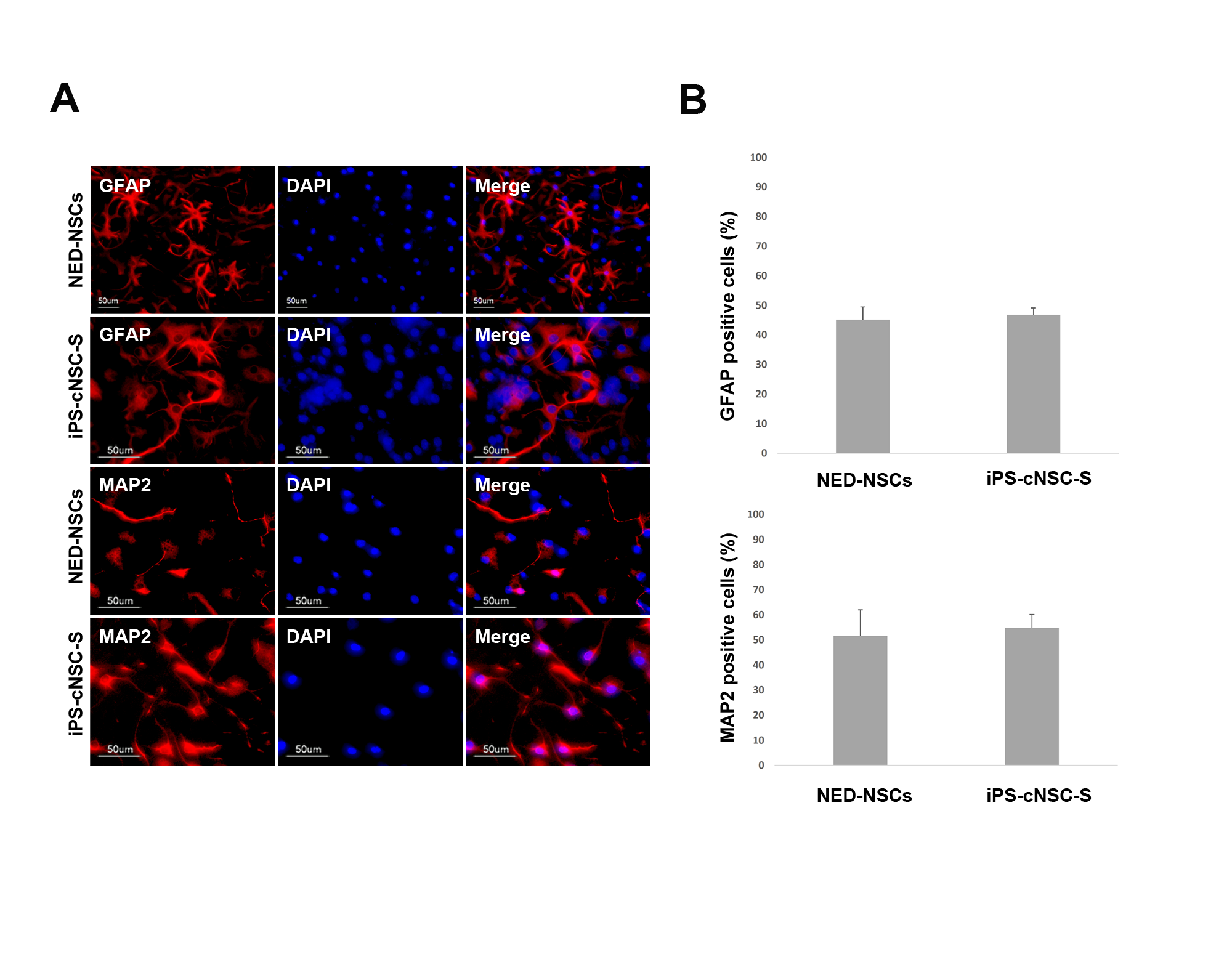

Supplement: S3 Fig — (A) iPS-cNSC-S and NED-NSCs could differentiate into glial (GFAP+) or neural (MAP2+) lineage. (B) Quantification of the lineage-specific differentiation efficiency by NED-NSCs and iPS-cNSC-S. Error bars indicate the standard error of the mean. (TIF) [file pone.0170735.s003.tif]

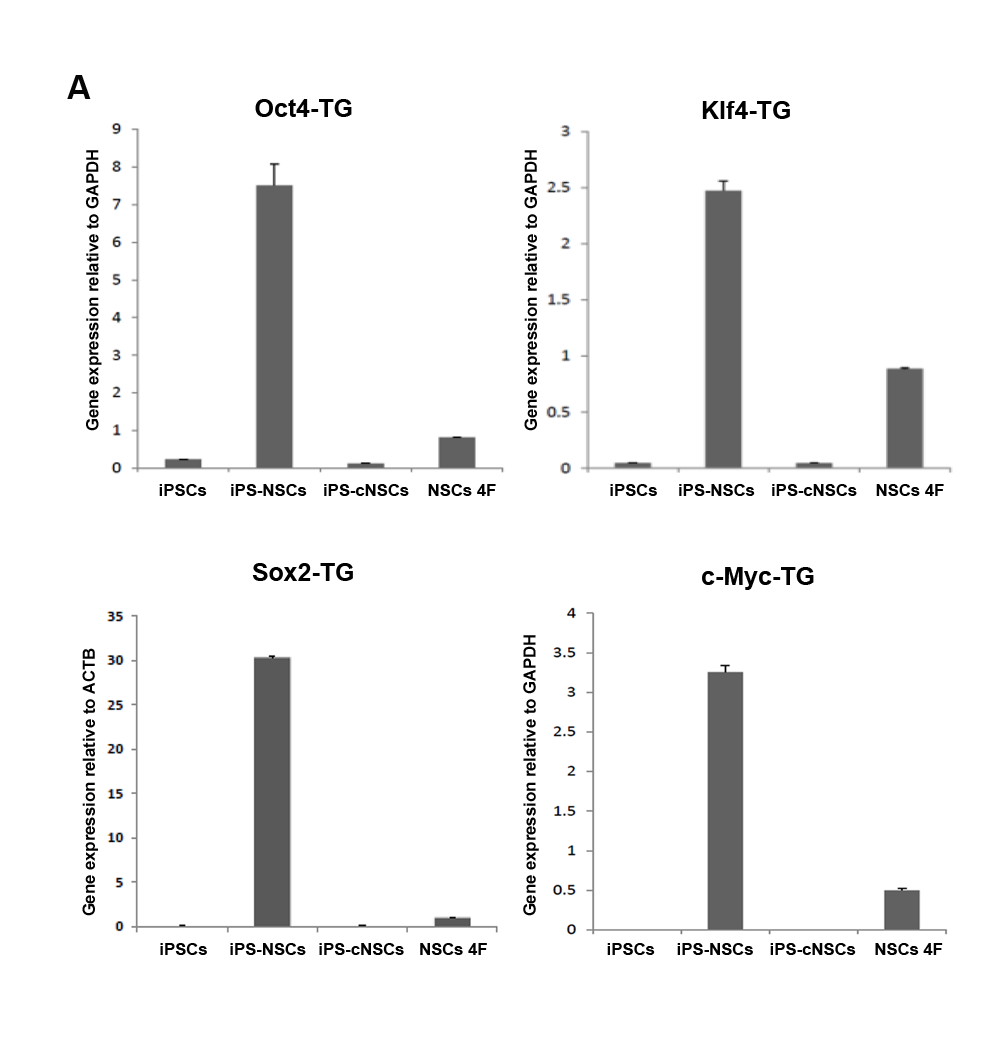

Supplement: S4 Fig — (A) Expression levels of exogenous 4 Factor (Oct4-TG, Klf4-TG, Sox2-TG, and c-Myc-TG) in iPSCs (Negative control), iPS-NSCs (Positive control, Transgene re-expressed, established in our previous article [14]), iPS-cNSCs, and NSCs 4F (Positive control). NSCs 4F were control, infected retroviral four factors in, respectively. Data are presented as mean±SD of triplicates (n = 3). (TIF) [file pone.0170735.s004.tif]

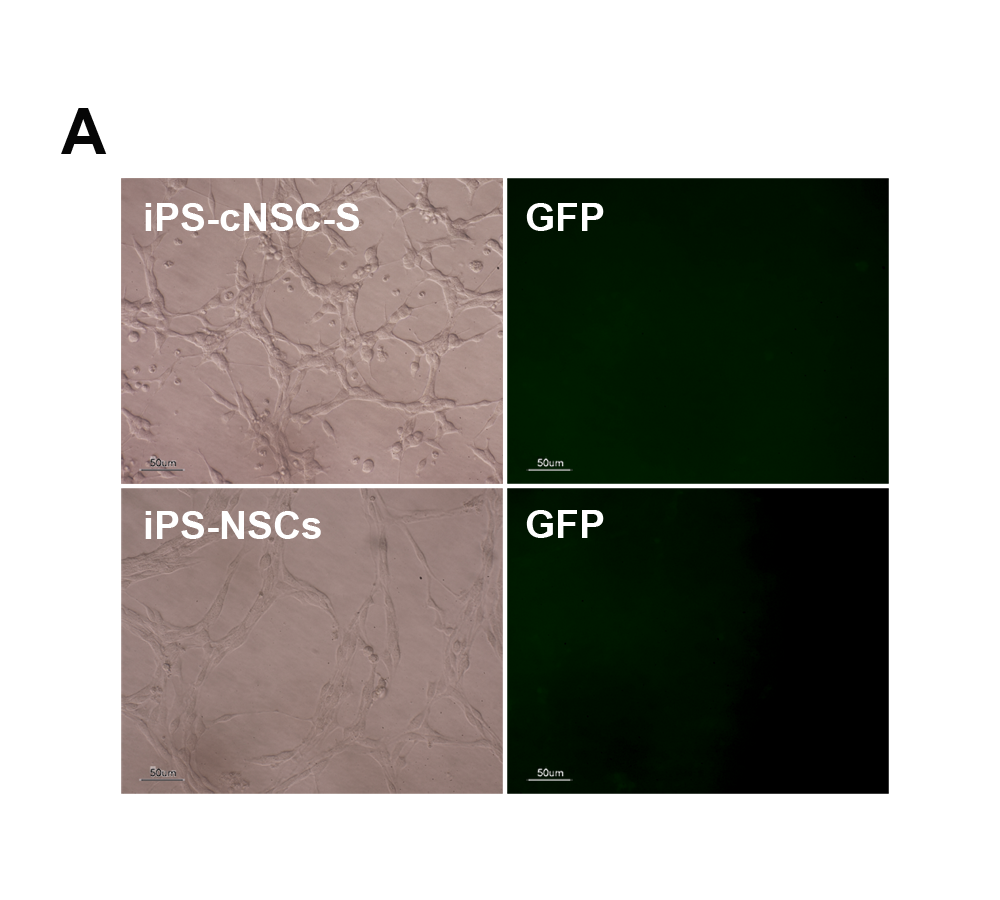

Supplement: S5 Fig — (A) iPS-cNSC-S and iPS-NSCs were negative for Oct4-GFP transgene expression. (TIF) [file pone.0170735.s005.tif]
