# Supplementary material for: In vivo differentiation of induced pluripotent stem cells into neural stem cells by chimera formation
Source: PLoS One. 2017 Jan 31;12(1):e0170735. doi: 10.1371/journal.pone.0170735 (PMC5283667; doi:10.1371/journal.pone.0170735)
Supplement: S1 Table — (PDF) [file pone.0170735.s006.pdf]

**Annotation Cluster 1**	Enrichment Score: 5.43	
**GO term**	**Count**	**P_Value**
blood vessel development	15	2.30E-06
vasculature development	15	3.10E-06
blood vessel morphogenesis	13	7.20E-06

**Annotation Cluster 2**	Enrichment Score: 2.47	
**GO term**	**Count**	**P_Value**
actin cytoskeleton organization	9	1.10E-03
actin filament-based process	9	1.70E-03
cytoskeleton organization	10	2.20E-02

**Annotation Cluster 3**	Enrichment Score: 1.93	
**GO term**	**Count**	**P_Value**
embryonic organ morphogenesis	8	4.10E-03
embryonic organ development	9	1.10E-02
embryonic morphogenesis	10	3.70E-02

**Annotation Cluster 4**	Enrichment Score: 1.9	
**GO term**	**Count**	**P_Value**
regulation of phosphorylation	10	1.10E-02
regulation of phosphorus metabolic process	10	1.40E-02
regulation of phosphate metabolic process	10	1.40E-02

**Annotation Cluster 5**	Enrichment Score: 1.81	
**GO term**	**Count**	**P_Value**
cholesterol biosynthetic process	4	2.80E-03
sterol biosynthetic process	4	6.10E-03
steroid biosynthetic process	5	1.20E-02
cholesterol metabolic process	4	5.70E-02
sterol metabolic process	4	7.20E-02

Annotation Cluster 6	Enrichment Score: 1.77	
GO term	Count	P_Value
membrane invagination	8	9.50E-03
endocytosis	8	9.50E-03
membrane organization	8	5.50E-02

**Annotation Cluster 7**	Enrichment Score: 1.47	
**GO term**	**Count**	**P_Value**
regulation of protein kinase activity	7	3.00E-02
regulation of kinase activity	7	3.40E-02
regulation of transferase activity	7	3.90E-02

**Annotation Cluster 1**	Enrichment Score: 1.26	
**KEGG pathway**	**Count**	**P_Value**
Small cell lung cancer	5	2.10E-02
ECM-receptor interaction	4	8.30E-02
Focal adhesion	6	9.70E-02

**Annotation Cluster 2**	Enrichment Score: 0.97	
**KEGG pathway**	**Count**	**P_Value**
Arrhythmogenic right ventricular cardiomyopathy	5	1.40E-02
Hypertrophic cardiomyopathy (HCM)	3	2.80E-01
Dilated cardiomyopathy	3	3.20E-01

**Annotation Cluster 1**	Enrichment Score: 3.07	
**GO term**	**Count**	**P_Value**
acetyl-CoA metabolic process	7	8.40E-06
coenzyme metabolic process	8	8.30E-03
cofactor metabolic process	9	9.10E-03

**Annotation Cluster 2**	Enrichment Score: 2.78	
**GO term**	**Count**	**P_Value**
cofactor catabolic process	6	1.40E-04
tricarboxylic acid cycle	5	4.50E-04
acetyl-CoA catabolic process	5	5.30E-04
aerobic respiration	5	8.50E-04
coenzyme catabolic process	5	1.10E-03
cellular respiration	5	1.50E-02
energy derivation by oxidation of organic compounds	5	7.30E-02

**Annotation Cluster 3**	Enrichment Score: 2.28	
**GO term**	**Count**	**P_Value**
nucleotide biosynthetic process	10	2.00E-03
ATP biosynthetic process	7	2.00E-03
nucleobase, nucleoside, nucleotide and nucleic acid biosynthetic process	10	2.40E-03
nucleobase, nucleoside and nucleotide biosynthetic process	10	2.40E-03
purine ribonucleotide metabolic process	8	3.10E-03
ATP metabolic process	7	3.20E-03
purine ribonucleoside triphosphate biosynthetic process	7	3.80E-03
ribonucleoside triphosphate biosynthetic process	7	3.80E-03
ribonucleotide metabolic process	8	4.00E-03
purine nucleoside triphosphate biosynthetic process	7	4.00E-03
nucleoside triphosphate biosynthetic process	7	4.20E-03
purine ribonucleoside triphosphate metabolic process	7	5.70E-03
ribonucleoside triphosphate metabolic process	7	6.00E-03
purine nucleotide biosynthetic process	8	6.60E-03
purine nucleoside triphosphate metabolic process	7	7.20E-03
purine ribonucleotide biosynthetic process	7	7.50E-03
ribonucleotide biosynthetic process	7	8.90E-03
nucleoside triphosphate metabolic process	7	1.10E-02
purine nucleotide metabolic process	8	1.50E-02
nitrogen compound biosynthetic process	10	5.50E-02

**Annotation Cluster 4**	Enrichment Score: 1.93	
**GO term**	**Count**	**P_Value**
regulation of transmission of nerve impulse	7	7.50E-03
regulation of neurological system process	7	9.70E-03
regulation of synaptic transmission	6	2.20E-02

**Annotation Cluster 5**	Enrichment Score: 1.87	
**GO term**	**Count**	**P_Value**
ion homeostasis	12	7.80E-03
cellular homeostasis	13	9.40E-03
cellular ion homeostasis	11	9.60E-03
cellular chemical homeostasis	11	1.10E-02
chemical homeostasis	13	1.50E-02
homeostatic process	16	4.90E-02

**Annotation Cluster 6**	Enrichment Score: 1.67	
**GO term**	**Count**	**P_Value**
regulation of nervous system development	8	9.90E-03
regulation of neurogenesis	7	2.00E-02
regulation of neuron differentiation	6	2.40E-02
regulation of cell development	7	4.30E-02

**Annotation Cluster 1**	Enrichment Score: 0.9	
**KEGG pathway**	**Count**	**P_Value**
Oxidative phosphorylation	7	4.40E-02
Alzheimer's disease	8	6.90E-02
Parkinson's disease	5	2.70E-01
Huntington's disease	6	3.00E-01
