# Supplementary material for: In vivo differentiation of induced pluripotent stem cells into neural stem cells by chimera formation
Source: PLoS One. 2017 Jan 31;12(1):e0170735. doi: 10.1371/journal.pone.0170735 (PMC5283667; doi:10.1371/journal.pone.0170735)
Supplement: S2 Table — (PDF) [file pone.0170735.s007.pdf]

**Annotation Cluster 1**	Enrichment Score: 1.89	
**GO term**	**Count**	**P_Value**
cell-cell adhesion	7	3.60E-03
cell adhesion	9	2.40E-02
biological adhesion	9	2.40E-02

**Annotation Cluster 2**	Enrichment Score: 1.81	
**GO term**	**Count**	**P_Value**
regulation of neurogenesis	5	9.40E-03
regulation of nervous system development	5	1.40E-02
regulation of cell development	5	1.80E-02
regulation of neuron differentiation	4	2.60E-02

**Annotation Cluster 1**	Enrichment Score: 4.11	
**GO term**	**Count**	**P_Value**
tube morphogenesis	10	3.80E-05
tube development	12	4.60E-05
epithelium development	11	2.70E-04

**Annotation Cluster 2**	Enrichment Score: 3.63	
**GO term**	**Count**	**P_Value**
epithelial tube morphogenesis	8	9.30E-05
morphogenesis of an epithelium	9	2.60E-04
epithelium development	11	2.70E-04
tissue morphogenesis	10	4.70E-04

**Annotation Cluster 3**	Enrichment Score: 3.33	
**GO term**	**Count**	**P_Value**
chordate embryonic development	14	2.00E-04
embryonic development ending in birth or egg hatching	14	2.20E-04
embryonic morphogenesis	11	2.40E-03

**Annotation Cluster 4**	Enrichment Score: 2.51	
**GO term**	**Count**	**P_Value**
primary neural tube formation	5	6.80E-04
neural tube formation	5	1.40E-03
embryonic epithelial tube formation	5	1.70E-03
tube lumen formation	5	2.40E-03
neural tube closure	4	6.10E-03
tube closure	4	6.10E-03
morphogenesis of embryonic epithelium	5	6.60E-03
neural tube development	5	8.20E-03

**Annotation Cluster 5**	Enrichment Score: 1.96	
**GO term**	**Count**	**P_Value**
embryonic skeletal system morphogenesis	5	3.30E-03
skeletal system morphogenesis	6	8.20E-03
embryonic skeletal system development	5	8.20E-03
embryonic organ morphogenesis	5	6.90E-02

**Annotation Cluster 6**	Enrichment Score: 1.84	
**GO term**	**Count**	**P_Value**
cell morphogenesis	9	9.70E-03
cell morphogenesis involved in differentiation	7	1.60E-02
cellular component morphogenesis	9	2.00E-02

**Annotation Cluster 7**	Enrichment Score: 1.52	
**GO term**	**Count**	**P_Value**
pyrimidine nucleotide biosynthetic process	4	8.70E-04
pyrimidine nucleotide metabolic process	4	2.10E-03
nucleotide biosynthetic process	5	8.60E-02
nucleobase, nucleoside and nucleotide biosynthetic process	5	9.30E-02
nucleobase, nucleoside, nucleotide and nucleic acid biosynthetic process	5	9.30E-02
nucleoside metabolic process	3	1.00E-01
nitrogen compound biosynthetic process	6	1.60E-01
